# Living on borrowed time – Amazonian trees use decade‐old storage carbon to survive for months after complete stem girdling

**DOI:** 10.1111/nph.15302

**Published:** 2018-08-01

**Authors:** Jan Muhr, Susan Trumbore, Niro Higuchi, Norbert Kunert

**Affiliations:** ^1^ Max‐Planck‐Institute for Biogeochemistry Hans‐Knöll‐Str. 10 Jena 07745 Germany; ^2^ Earth System Science University of California Irvine Irvine CA 92697‐3100 USA; ^3^ Laboratory of Forest Management Brazilian National Institute for Research in the Amazon Manaus Brazil; ^4^ Conservation Ecology Center Smithsonian Conservation Biology Institute 1500 Remount Rd. MRC 5535 Front Royal VA 22630 USA; ^5^ Center for Tropical Forest Science‐Forest Global Earth Observatory Smithsonian Tropical Research Institute Panama City Panama

**Keywords:** carbon reserves, nonstructural carbohydrates, radiocarbon, tree girdling, tree survival

## Abstract

- Nonstructural carbon (NSC) reserves act as buffers to sustain tree activity during periods when carbon (C) assimilation does not meet C demand, but little is known about their age and accessibility; we designed a controlled girdling experiment in the Amazon to study tree survival on NSC reserves.
- We used bomb‐radiocarbon (^14^C) to monitor the time elapsed between C fixation and release (‘age’ of substrates). We simultaneously monitored how the mobilization of reserve C affected δ^13^ CO_2_.
- Six ungirdled control trees relied almost exclusively on recent assimilates throughout the 17 months of measurement. The Δ^14^C of CO_2_ emitted from the six girdled stems increased significantly over time after girdling, indicating substantial remobilization of storage NSC fixed up to 13–14 yr previously. This remobilization was not accompanied by a consistent change in observed δ^13^ CO_2_.
- These trees have access to storage pools integrating C accumulated over more than a decade. Remobilization follows a very clear reverse chronological mobilization with younger reserve pools being mobilized first. The lack of a shift in the δ^13^ CO_2_ might indicate a constant contribution of starch hydrolysis to the soluble sugar pool even outside pronounced stress periods (regular mixing).

## Introduction

Trees can allocate carbon (C) to structural biomass, which is usually considered immobile and no longer accessible, or to mobile nonstructural carbon (NSC). Some NSC serves as an immediate source of C and energy (‘transient NSC pool’), but substantial amounts also can be stored for longer time periods (‘reserve NSC’) in perennial organs (branches, roots, stem) (Hoch *et al*., 2003). Questions concerning the turnover time of reserve NSC, its accessibility to the tree over time and its role in tree metabolism are still largely unresolved (Dietze *et al*., 2014), but highly important when trying to predict a tree's resilience to C starvation (McDowell *et al*., 2011). Generally, reserve NSC serves as a buffer for periods when a tree's C assimilation is inadequate to meet its C demand. Trees from several biomes have been shown to access reserves that are several years old following massive disturbance, for example when forced to regrow roots after hurricane damage (Vargas *et al*., 2009), growing stump sprouts after the stem has been cut down (Carbone *et al*., 2013), or during winter in the absence of assimilation, as shown for Canadian sugar maple (Muhr *et al*., 2016). There also is growing evidence that older reserve NSC can even be mobilized during periods that are not linked to stress or a lack of C supply (Schuur & Trumbore, 2006; Muhr *et al*., 2013), suggesting that reserve NSC is regularly mixed with more recently assimilated C pools (Richardson *et al*., 2013).

The mean age of C – the mean time elapsed since the constituent C in NSC or structural C was fixed from the atmosphere – can be estimated using the known history of bomb‐radiocarbon in the atmosphere. Thermonuclear weapon testing in the late 1950s and early 1960s caused a rapid increase of the radiocarbon signature (Δ^14^C) of atmospheric CO_2_ (Trumbore *et al*., 2016). With the nuclear test ban treaty ending most atmospheric tests in 1964, atmospheric Δ^14^CO_2_ concentrations declined over the subsequent decades as excess ^14^C was taken up by terrestrial and oceanic C reservoirs and diluted with ^14^C‐free CO_2_ originating from fossil fuel combustion (Levin & Hesshaimer, 2000). Each year's atmospheric Δ^14^CO_2_ signature propagates directly into plant biomass through photosynthetic uptake. Comparing the Δ^14^C of tree C pools with the atmospheric record thus can yield information on the average time elapsed since constituent C was fixed from the atmosphere, thus allowing, for example, the mobilization of previously accumulated reserve C to be monitored.

Starch usually is ^13^C enriched compared to photosynthetic sugars (Brugnoli *et al*., 1988; Eglin *et al*., 2009; Maunoury‐Danger *et al*., 2010), and sugars produced from starch hydrolysis also are expected to be ^13^C‐enriched. Assuming that respiration in the stem of an unstressed tree is fueled mainly by photosynthetic sugars imported *via* the phloem, the increasing respiration of sugars derived from starch hydrolysis might increase the δ^13^CO_2_ as reported for 2‐yr‐old sessile oak sapling affected by girdling (Maunoury‐Danger *et al*., 2010).

Here, we report data from a girdling experiment (including a control group) conducted on a hyperdominant (*c*. five individuals per hectare) tree in a central Amazon tropical forest north of Manaus, Brazil. Girdling – that is, the complete circumferential removal of bark, phloem and cambium near the base of a tree stem – results in an immediate shut‐down of the supply of new assimilates from the canopy and thus in the death of the area below the girdling and eventually the whole tree. Due to the complete termination of C supply from the canopy, any metabolism below the girdle must be fueled by C that is already present, that is, the tree is forced into mobilizing reserve NSC. To our knowledge, this is the first experiment that forces mature trees into mobilization of reserve C for a prolonged period while using bomb‐radiocarbon to actually measure the age of the accessed reserves. We were especially interested to learn how fast the trees would have to mobilize reserves clearly older than recently assimilated C, how this mobilization would progress over time, and whether we could identify a maximum age of mobilized reserves, helping to understand over which periods trees accumulate reserves and can keep them accessible.

## Materials and Methods

We selected 12 *Scleronema micranthum* (Ducke) Ducke individuals within the Estação Experimental de Silvicultura Tropical (EEST), a 21 000 ha reserve of the Instituto Nacional de Pesquisas da Amazônia (INPA) ZF2 experimental forest north of Manaus, Brazil, located in a well‐drained tropical moist *terra firme* forest *c*. 60 km northwest of Manaus, Brazil (02°38′22.54″S 60°09′51.34″W). The trees were divided in a control and a girdling group with similar diameters at breast height (mean dbh ± SD of 41.2 ± 13.3 and 41.1 ± 7.5 cm, respectively) and were free of disease, critical injuries or any obvious signs of dieback. Mean annual temperature is 25.8°C and annual rainfall averages 2550 mm. Between June and October (dry season), monthly rainfall can drop below 100 mm (Kunert *et al*., 2015). First samples were collected in 2012 on a subset of seven trees. These data were mainly for validating the sampling method and estimating seasonal variability. In April 2013 we sampled CO_2_ formed at different depths (0, 4, 8 and 12 cm) within the stem from these seven initial trees. At the same time we started pre‐girdling measurements on all 12 individuals. On 3 October 2013, six of the trees were girdled at a height between 1.5 and 1.8 m above ground. The ^14^C and ^13^C signature of stem CO_2_ efflux and in‐stem CO_2_ from tree stems below the girdling height as well as the six ungirdled control trees were measured at intervals until August 2014, when two of the girdled trees were clearly dying and under insect attack by the metallic wood boring beetle (*Euchroma gigantea,* Buprestidae).

### CO_2_ sampling

For CO_2_ sampling, we used (1) incubation chambers temporarily installed on the stem surface, (2) permanently installed in‐stem gas probes, and (3) laboratory incubations of extracted live stem cores. Incubation chambers and in‐stem gas probes at 4 cm depth were sampled across the whole study period. Additional in‐stem gas probes inserted to further depths of 8 and 12 cm were installed only in seven trees and sampled before girdling. Live stem cores were extracted only in March, May and August of 2014, toward the end of the experiment. Independent of the measurement, gas samples containing CO_2_ derived from chambers, probes or incubations were taken using custom‐built glass flasks (volume: 45 or 115 ml) equipped with an O‐ring valve (Louwers H.V. glass valves, Louwers Glass and Ceramic Technologies, Hapert, Netherlands; 12 mm OD, 9 mm ID) and shipped to the laboratory for further analysis.

Stem chambers were built from 15‐cm‐long pieces of polypropylene tubing (6.5 cm OD) that were welded shut on both sides with polypropylene discs. By cutting off a segment (height 2 cm) the tube was turned into a cuvette isolating an approximate air volume of 260 ml over the stem surface. Chambers were covered with self‐adhesive aluminum foil to reduce warming by solar radiation. Chambers were equipped with three fittings (Sprint ESKV 20; Wiska Hoppmann GmbH, Kaltenkirchen, Germany) sealed with liquid rubber around the edges (Dichtfix; Bindulin, Fürth, Germany). For sampling, lichens and mosses were carefully removed from the installation location and the chambers were attached to the trees with four lashing straps. To achieve a gastight seal, a frame (25 mm thick, outer dimensions: 20.5 cm long, 9.5 cm wide, inner dimensions: 14 cm long, 4.5 cm wide) made from closed‐porous cellular rubber (EPDM‐quality; Reiff Technische Produkte GmbH, Reutlingen, Germany) was placed between the chamber and the bark (the bark was very thin and smooth on this species, so no additional pre‐treatment was necessary). Chambers were considered leak‐tight when blowing respiratory air through a tube along the edges did not result in increasing [CO_2_] inside the chamber measured with a portable infrared gas analyzer system featuring a Li‐820 (Li‐Cor Environmental GmbH, Bad Homburg, Germany). Up to three flasks were connected to the chamber after installation, opened and then left for equilibration for 4–7 d before they were closed and chambers were removed from the stem.

In‐stem gas probes were stainless steel tubes (OD 12.7 mm) permanently installed in the tree stems at various sampling depths that could be equipped with up to three flasks simultaneously. After drilling a 12‐mm‐wide hole at breast height (drill hole depth 4, 8 or 12 cm), a piece of stainless steel tubing (length 9, 13 or 17 cm, OD 12.7 mm) was hammered into the stem and sealed around the edges with hot glue. For the control trees, we placed the three different in‐stem gas probes 8 cm horizontally distant from each other at the same height. After installation and in between sampling periods, the protruding 5 cm of tubing were closed using silicone protective caps (11.2 mm diameter, Versilic; Saint‐Gobain Performance Plastics, Charny, France) to avoid infestation with insects or fungi or continuous gas exchange with the atmosphere. For sampling, we attached a stainless steel T‐ or cross piece (12.7 mm ID, Swagelok; B.E.S.T. Fluidsysteme GmbH, Leipzig, Germany) with Teflon nuts and ferrules that could hold either two or three sampling flasks, respectively. Attached flasks were opened and then allowed to equilibrate with the stem‐internal gas phase for 4–7 d. Note that over time, stem growth inside the gas probes (wound response) fully sealed the contact surface, making sampling impossible. Therefore, drill holes in the control trees (which had been originally drilled 1 yr earlier than the girdling trees) had to be re‐drilled in March 2014, and sampling attempts between September 2013 and March 2014 failed for the control trees until we identified the problem.

Incubations of live stem cores were added later to the experiment as a means to measure *in situ* CO_2_ production free from transport and diffusion effects. Live stem cores were incubated in custom‐built gas‐tight incubation cylinders made of plexiglass (length 8.5 cm, inner diameter 2 cm) equipped with a lid sealed by a Viton O‐Ring on each side. Lids were equipped with fittings (12.7 mm Swagelok Ultra‐Torr; B.E.S.T. Fluidsysteme GmbH), for attaching an open sampling flask on each side. We extracted live stem cores using an increment corer (core diameter 5.15 mm; Haglöf Sweden AB, Långsele, Sweden), then removed the bark and cut the cores to a length of 6 cm. Before starting the incubation, cores could equilibrate with the atmosphere for 6–8 h, while continually being kept moist. The incubation volume and the flasks were then flushed with CO_2_‐free air and the incubation was started. The incubation cylinders were left at room temperature for 24 h while CO_2_ was allowed to accumulate; subsequently the flask valves were closed.

### Processing and measurement of samples

Dried biomass samples were ground and then combusted in the presence of CuO in a pre‐combusted and evacuated Quartz tube at 900°C. For radiocarbon analysis, CO_2_ from combusted biomass samples and from sampling flasks was cryogenically purified and converted to graphite targets using the modified sealed tube zinc reduction method described by Xu *et al*. (2007). All graphitized samples were analyzed by the Keck Carbon Cycle AMS facility at University of California, Irvine with a precision and accuracy of 2–3‰ (Xu *et al*., 2007). Radiocarbon data are expressed as Δ^14^C, which is the per mil deviation from the ^14^C/^12^C ratio of oxalic acid standard in 1950. The sample ^14^C/^12^C ratio is corrected to a δ^13^C value of −25‰ to account for any mass‐dependent fractionation effects (Stuiver & Polach, 1977). Thus, our Δ^14^C values can be directly compared with the record of ^14^C in atmospheric CO_2_ for the Southern Hemisphere.

For measurements of δ^13^CO_2_, we separated a small subsample during processing of the radiocarbon samples. Following purification and before graphitization we transferred 20–40 μl of CO_2_ with a gas‐tight syringe (Hamilton 1750 SL; Hamilton Co., Reno, NV, USA) into a septum‐capped 12‐ml flat‐bottomed Soda Glass Vial (Labco Exetainer; Labco Ltd, Lampeter, UK) pre‐flushed with CO_2_‐free N_2_. The δ^13^C isotopic signature of CO_2_ in air was analyzed using an isotope ratio mass spectrometer (Delta^+^ XL; Thermo Fisher Scientific, Bremen, Germany) coupled to a modified gas bench via a Conflow III and GC (Thermo Fisher Scientific). Aliquots of samples were injected into the GC using a CTC Combi‐PAL autosampler. The CO_2_ was baseline‐separated from the other air constituents using a 30 m Poraplot Q column, and water was removed using two Nafion traps and a dry ice/ethanol trap. Samples were analyzed against a laboratory air standard thus satisfying the principle of identical treatment (Werner *et al*., 2001). δ^13^C values are reported on the VPDB scale realized by the Jena Reference Air Set‐06 (JRAS‐06) scale for isotopes of CO_2_ in air (Wendeberg *et al*., 2013).

### Estimating the ^14^C‐based C age

We compared Δ^14^C of samples to the atmospheric Δ^14^C to estimate the time elapsed since fixation by the tree in two different ways. First, we averaged annual atmospheric Δ^14^C based on the southern hemisphere atmospheric record published by Hua *et al*. (2013) for the years 1950–2011 and combined the data with the Δ^14^C of monitoring plant biomass sampled at our site between 2012 and 2014, then calculated the mean annual decline of atmospheric Δ^14^C. Differences in sample Δ^14^C and atmospheric Δ^14^C then can be translated into an average age based on this mean annual decline. Alternatively, we used a steady‐state one‐pool turnover time model as described in Gaudinski *et al*. (2001) to estimate the Δ^14^C that would be expected for a C pool in the year 2014 when assuming C input as described by the atmospheric record of Hua *et al*. (2013) for 1950–2011 and varying the turnover time between 2 and 20 yr, and compared these values to the sample Δ^14^C. The two methods should yield similar results for C pools < 20 yr old.

Note that it is technically impossible to apply these methods to samples with Δ^14^C values significantly below the current atmospheric background, that is, samples affected by pre‐bomb CO_2_. These five samples (out of 310) were removed from the dataset before analysis.

### Statistics and number of sampled trees

We started with a total of seven individuals in 2012, 18 months before the girdling (*n *=* *7 per campaign). We decided to increase the number of individuals from 7 to 12 in April 2013, at the same time defining two groups for the purpose of the girdling experiment (control and girdling group, *n *=* *6 each). However, due to uncontrollable losses of glass flasks during shipment the number of replicates for each of the two groups occasionally was less than six.

We checked the data for significant differences from normal distribution using the Shapiro–Wilk test. For normally distributed data we tested for differences using the Student's *t*‐test (two‐sided, nonpaired, unequal variance). For data with non‐normal distribution we instead used the nonparametric Wilcoxon–Mann–Whitney test (WMW). We tested for significant differences between control and girdling trees for simultaneous measurements (^14^C, ^13^C) and between atmospheric background and sample data (^14^C).

In addition, we tested for significant differences between samples from different depths along an in‐stem profile in the control trees (^14^C and ^13^C). This was done using a Kruskal–Wallis rank sum test followed by pairwise comparison of individual depths using the WMW. All statistical analyses were performed in the R statistical environment (R Development Core Team, 2008; v3.3.1) using RStudio (v.1.0.136) (RStudioTeam, 2015).

## Results

### Pre‐experimental Δ^14^C and δ^13^C data

**Figure 1:**
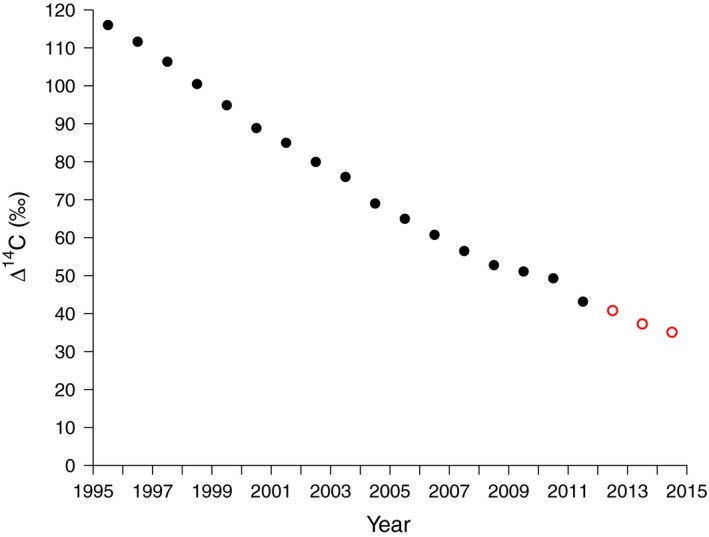
Mean Δ^14^C of the southern hemisphere atmospheric record (solid black circles) as published by Hua *et al*. (2013) and of local monitoring plant biomass (open red circles).

The Δ^14^C of atmospheric CO_2_ declined at a rate of 4.3‰ yr^−1^ (*r* ^2^ = 0.99) during our experiment (Fig. 1). This rate of decline is greater than the accuracy of our ^14^C measurements (2–3‰), thus allowing us to use the bomb‐radiocarbon approach as described in the methods.

In 2012, chamber Δ^14^CO_2_ revealed no significant differences between wet (47 ± 3; *n *=* *7) and dry season (43 ± 1‰; *n *=* *7) (data not shown) with the overall average (45 ± 2‰, *n *=* *14) being close to the atmospheric background derived from annual plants growing in 2012, 41 ± 0‰ (*n *=* *2; Fig. 1), which we assumed recorded the Δ^14^C of recently fixed C; pre‐experimental variability (both seasonal and between trees) thus was considered negligible for a girdling experiment.

**Figure 2:**
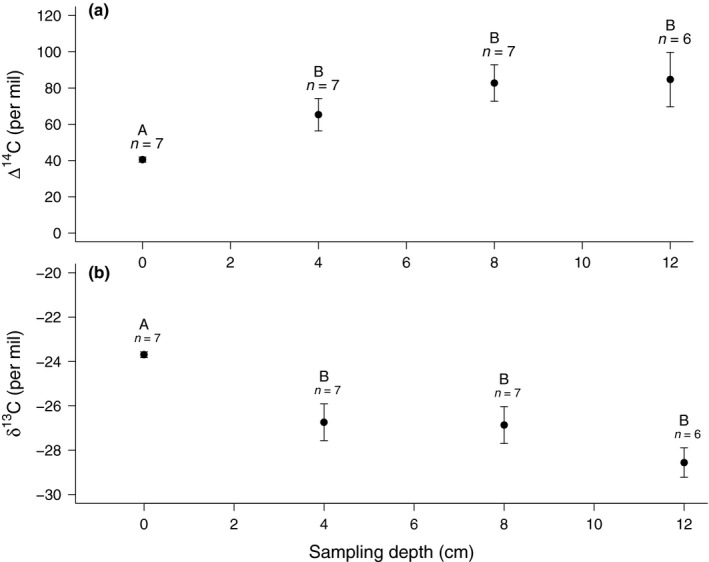
Mean (± SE) values of (a) Δ^14^C and (b) δ^13^C of CO_2_ measured at different sampling depths in the stems of the original seven *Scleronema micranthum* trees (with 0 cm depth being equal to emissions at the stem surface) in April 2013. Statistically significant differences (*P *<* *0.05) between sampling depth averages are indicated by different letters. The number of replicates (*n*) is seven for sampling depths 0, 4 and 8 cm, but only *n *=* *6 for 12 cm due to sample loss during transport.

The Δ^14^C of tree internal CO_2_ pools sampled by in‐stem probes in April 2013 was significantly higher than the 41 ± 1‰ in the stem surface emissions, and increased with depth in the stem from 66 ± 9‰ (*n *=* *7) at 4 cm to 83 ± 10‰ (*n *=* *7) at 8 cm, and 85 ± 15‰ (*n *=* *6) at 12 cm depth (Fig. 2), thus making lateral transport of CO_2_ a possible source for enriched Δ^14^CO_2_, which is why we included the additional live stem core incubations. Concentrations of CO_2_ were high inside the stem, with mean equilibrium concentrations of 0.7, 3.4 and 2.2% CO_2_ at 4, 8 and 12 cm depths, respectively. The δ^13^C of CO_2_ declined from −23.7 ± 0.1‰ (*n* = 7) at a depth of 0 cm (chamber headspace), to −26.7 ± 0.8 (*n *=* *7), −26.9 ± 0.8 (*n *=* *7) and −28.6 ± 0.7‰ (*n *=* *6) at 4, 8 and 12 cm depth, respectively.

### Effect of the girdling on Δ^14^CO_2_ and δ^13^CO_2_

**Figure 3:**
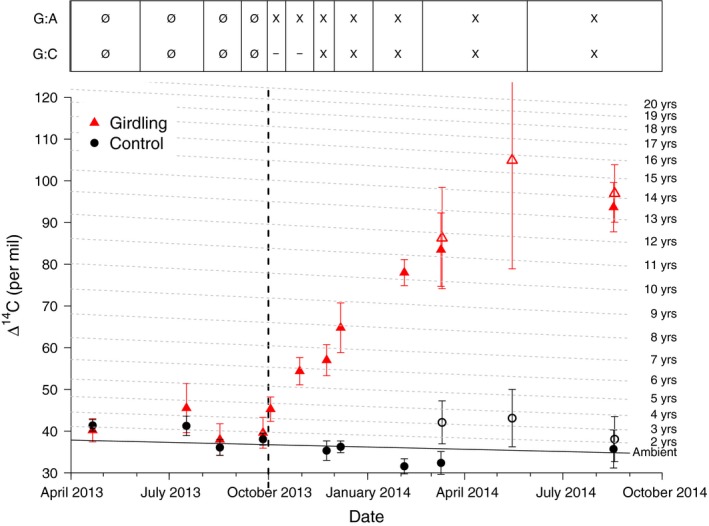
Mean Δ^14^C (± SE) of the chamber (closed symbols) and stem core (open symbols) incubation samples of the control (black circles) and girdled (red triangles) *Scleronema micranthum* trees. The time of girdling is indicated by the vertical dashed line. The atmospheric Δ^14^C is indicated by the solid horizontal line (‘Ambient’). The dashed horizontal lines indicate the expected Δ^14^C at a given time for a pool with a turnover time as indicated on the right side of the graph and was calculated based on the one‐pool, steady‐state model by Gaudinski *et al*. (2001). The top panel shows when we found statistically significant differences between the girdling and control treatment (G : C) or the girdling treatment compared to the atmospheric background (G : A) (statistics for chamber data only, not for stem core incubations). ‘X’ and ‘Ø’ indicate that data were, or were not, statistically significant different (*P *<* *0.05 or *P *≥* *0.05), respectively, whereas ‘–’ indicates that no test was performed.

The mean pre‐girdling Δ^14^CO_2_ (± SE) of stem‐emitted CO_2_ measured over four subsequent campaigns was 39 ± 1‰ (*n *=* *24) and 41 ± 2‰ (*n *=* *23) for control and girdled groups, respectively, not significantly different (WMW, *P *>* *0.05) from the Δ^14^C of C fixed and recorded in annual plant tissues in 2013 (37 ± 2‰, *n *=* *6) (Fig. 3). For control trees, the Δ^14^CO_2_ of CO_2_ that accumulated in stem chambers remained between 30‰ and 40‰ for the rest of the experiment. By contrast, the Δ^14^CO_2_ emitted from the stems of girdled trees steadily increased over subsequent months, from 41 ± 4‰ immediately before the girdling (September 2013) to a maximum of 94 ± 6‰ at the end of the experiment (August 2014), and was significantly higher than the background and control trees on all post‐girdling measurement dates (Fig. 3). The observed maximum (94 ± 6‰) was equivalent to the atmospheric Δ^14^CO_2_ in 1999 or the expected Δ^14^CO_2_ from a homogeneous pool with a turnover time of 13–14 yr.

**Figure 4:**
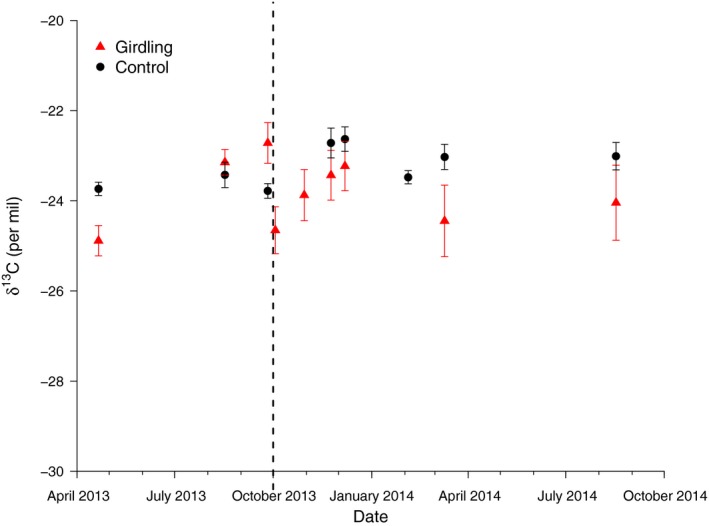
Mean δ^13^C (± SE) of the chamber incubation samples of control (black circles) and girdled (red triangles) *Scleronema micranthum* trees. The time of girdling is indicated by the vertical dashed line.

The mean δ^13^C values of CO_2_ in the chamber headspace ranged between −22‰ and −25‰ for both control and girdled trees throughout the measurement period (Fig. 4). Significant differences between the two groups did occur on individual dates, but they were inconsistent with the treatment.

**Figure 5:**
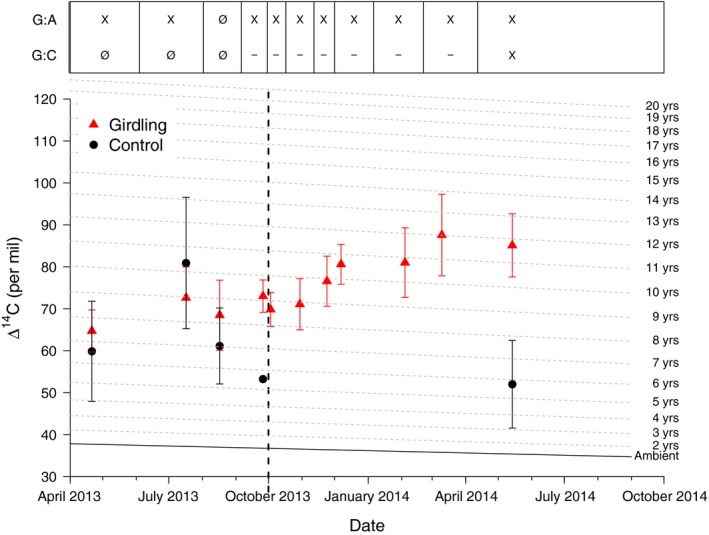
Mean Δ^14^C (± SE) of the in‐stem CO_2_ samples of the control (black circles) and girdled (red triangles) *Scleronema micranthum* trees. The time of girdling is indicated by the vertical dashed line. The atmospheric Δ^14^C is indicated by the solid horizontal line (‘Ambient’). The dashed horizontal lines indicate the expected Δ^14^C at a given time for a pool with a turnover time as indicated on the right side of the graph and was calculated based on the one‐pool, steady‐state model by Gaudinski *et al*. (2001). Control trees were not measured continuously. The top panel shows when we found statistically significant differences between the girdling and control treatment (G : C) or the girdling treatment compared to the atmospheric background (G : A). ‘X’ and ‘Ø’ indicate that data were, or were not, statistically significant different (*P *<* *0.05 or *P *≥* *0.05), respectively, whereas ‘–’ indicates that no test was performed.

Measurements of in‐stem probes are not available for all dates and depths for the whole experiment, due to clogging of the tubes over time. As this clogging occurred slowly, it only affected the in‐stem probes that were installed first (in April 2012). As all of the control trees happened to be among these, the clogging resulted in a loss of in‐stem data for the control group between October 2013 and May 2014. Pre‐girdling Δ^14^CO_2_ sampled at 4 cm depths in the stem were significantly higher than the atmospheric background on most dates with values ranging between 50‰ and 70‰ (Fig. 5). Girdling increased the mean Δ^14^CO_2_ up to 85 ± 8‰, which equals the atmospheric Δ^14^CO_2_ in 2000 and would be expected for a pool with a turnover time of 12–13 yr. This is significantly greater than the 52 ± 10‰ measured at the same depth in control trees in May 2014. Mean in‐stem Δ^14^CO_2_ of the girdled trees was significantly higher than atmospheric Δ^14^CO_2_ throughout the whole post‐girdling period. The in‐stem δ^13^C of the girdled trees remained constantly between −23.9‰ and −25.3‰ throughout the whole experiment and did not reveal differences consistent with the treatment (data not shown).

The mean Δ^14^CO_2_ of incubated live stem cores was greater (*P* ≤ 0.056) for cores extracted from girdled vs control trees (Fig. 3; Table 1). The Δ^14^CO_2_ of the control trees was very close to background atmospheric Δ^14^CO_2_, whereas the Δ^14^CO_2_ of the girdled trees was like that of the CO_2_ that accumulated in stem chamber headspace. These values are equivalent to atmospheric Δ^14^CO_2_ of the years 1997–2001, or to the Δ^14^CO_2_ of pools with turnover times between 12 and 17 yr, and track well with the Δ^14^C of CO_2_ emitted from the stems at the same time (Fig. 3).

**Table 1:** Mean Δ^14^C and mean equilibrium CO_2_ concentration of incubated live stem cores extracted from control and girdled *Scleronema micranthum* trees on three different dates

Date (d/month/yr)	Mean Δ^14^C ± SE (‰)			c(CO_2_) ± SE (%)		
	Girdling	Control	*P*	Girdling	Control	*P*
10/03/2014	86 ± 12	42 ± 5	0.029	1.9 ± 0.4	1.4 ± 0.3	0.5
14/05/2014	105 ± 26	43 ± 7	0.056	1.3 ± 0.3	1.3 ± 0.1	1.00
17/08/2014	97 ± 7	38 ± 5	0.004	0.9 ± 0.2	1.3 ± 0.2	0.3

The *P*‐values are given to indicate significant differences (*P *<* *0.05, Wilcoxon–Mann–Whitney *U‐*test) between the girdling and control data for each sampling date.

## Discussion

The increase of the mean Δ^14^CO_2_ – and therefore the mean age – of CO_2_ produced in, and emitted from tree stems following girdling demonstrates conclusively that the trees in this study not only have access to nonstructural carbon (NSC) reserves assimilated more than a decade previously, but also start using these old reserves to maintain metabolic activity as soon as the supply of new assimilates from the canopy is cut off. Trees not subjected to girdling preferentially use newly assimilated carbon (C) and it is the predominant source of CO_2_ emitted from the stem. The gradual increase in Δ^14^C of CO_2_ respired by stemwood and emitted from tree stems in the girdled trees suggests a reverse chronological mobilization of reserve NSC, with more recently assimilated C used first. Observations of increases in the age of NSC with depth in the stems of temperate forest trees (Richardson *et al*., 2015; Trumbore *et al*., 2015) suggest that access to older reserves to fuel metabolism is controlled by the spatial distribution of NSC pools within the stem.

The use of old reserves as ‘emergency’ C sources is in accord with other studies reporting the age of C mobilized by trees in reaction to massive disturbance, including C used for regrowing roots after massive hurricane damage (Vargas *et al*., 2009) or growing stump sprouts after the stem has been cut down (Carbone *et al*., 2013). Like girdling, both of these are situations in which mature trees are forced into covering metabolic C needs without any C supply from current assimilation. These two studies found maximum ages of mobilized C between 10 and 17 yr, which is in accord with the 13–14 yr we found toward the end of the experiment (Figs 3, 5). All trees in our experiment are estimated to be older than 100 yr based on two observations: First, we measured Δ^14^C of cellulose samples extracted from 8‐cm stem depth from four of the trees. All four samples were formed from pre‐bomb C, that is, C assimilated before the 1950s, suggesting average growth rates of < 1 mm yr^−1^. Second, tree diameters of our trees ranged between 27 and 58 cm (data not shown). Maximum ages of accessible reserve C could be affected by the sapwood lifespan, that is, the time that sapwood is alive and functional before it undergoes heartwood transformation. Average sapwood lifespans for various temperate and tropical tree species can vary tremendously between 8 and > 50 yr (Yang & Hazenberg, 1991; Spicer & Holbrook, 2007b; van der Sande *et al*., 2015). Assuming that sapwood contains reserves with an age similar to the year of its formation, this could explain the observed maximum ages of accessible reserves. In that context, we want to emphasize that ages estimated from Δ^14^C data represent averages of a nonsymmetrical distribution, that is, the actual pool contributing to CO_2_ production usually consists of a mix of C both younger and older than the estimated age, with the older C usually representing the smaller portion of the mix. Although it is impossible to determine the exact composition of this mixture, it thus can be assumed that a small proportion of the C contributing to respiration in our trees is several years, potentially even more than a decade, older than the observed average age.

Although the radiocarbon data clearly indicated a mobilization of reserve C following girdling, this was not accompanied by a respective change of the δ^13^C of the collected CO_2_, as significant differences occurred inconsistently both before and after the girdling. It has been reported before that stem δ^13^CO_2_ can vary throughout the season by up to several per mil (Maunoury *et al*., 2007), which is similar to the variation we see. Still, the lack of a clear effect of girdling on δ^13^CO_2_ was in contrast to our original expectations: Before girdling, the main source for local respiration in tree stems usually is expected to be phloem‐transported soluble sugars that were assimilated recently and then transported from source to sink organs. With phloem transport interrupted by girdling, the trees would have to release additional soluble sugars through the mobilization of reserve NSC (presumably through starch hydrolysis, although some trees have been reported to store lipids). As mentioned already, starch in woody organs usually is enriched in δ^13^C compared to photosynthetic sugars (Eglin *et al*., 2009), and we expected sugars derived from starch hydrolysis also to be enriched compared to the photosynthetic sugars, thus resulting in an increase of the δ^13^CO_2_ as soon as more of the respired sugars originated from starch hydrolysis. Such an increase has been reported after girdling on 2‐yr‐old sessile oak saplings (Maunoury‐Danger *et al*., 2010), where girdling resulted in an enrichment of δ^13^C of respired CO_2_ of *c*. 2–3‰ compared to the control trees. We did not see such an increase, however, and with the currently available data we can only speculate about the reason. One possible explanation that we would like to investigate further in the future is based on an observation reported by Richardson *et al*. (2013), who found Δ^14^C of starch and soluble sugars extracted from various depths of the tree stem of mature trees to be almost identical, thus making a continuous mixing of these two pools very likely. This would suggest that a portion of the soluble sugars in a mature tree stem always is derived from starch hydrolysis, even before girdling, and hence already ^13^C‐enriched compared to photosynthetic sugars in a leaf. The bigger the pre‐girdling contribution of starch‐derived sugars to the total soluble sugar pool in the stem, the less likely we would see an effect of the girdling on the δ^13^CO_2_.

Our pre‐experimental measurements showed that CO_2_ derived from decade‐old C sources can be found deeper within the stem of an unstressed tree. Although a large gradient exists between the free atmosphere (*c*. 0.04% CO_2_) and inside the stem, where CO_2_ concentrations averaged 2–3%, large differences in the isotopic signatures of CO_2_ (Fig. 2) indicate that these internal CO_2_ pools contribute little to stem surface emissions under normal conditions. The average Δ^14^CO_2_ at 8 cm depth was 86‰, that is, 48‰ higher than the atmospheric background, which would be expected for a pool with an average turnover time of 12–14 yr and reflects respiration from decade‐old C sources. Using a two‐pool mixing model (Phillips & Gregg, 2001) and the highly simplified assumption of two isotopically distinct endmembers (one being current assimilates, that is, with a Δ^14^CO_2_ equal to that years’ atmosphere, the other one equal to the Δ^14^CO_2_ pool at 8 cm), we estimate that the old pool contributed only *c*. 8% of the C emitted from the stem surface. Old CO_2_ also has been found inside live tree stems for three different tropical tree species in the Peruvian Amazon (Muhr *et al*., 2013). However, in that study older in‐stem pools contributed up to *c*. 20% of the CO_2_ emitted from tree stems.

It is well known that the part of the secondary xylem in tree stems that has not yet undergone heartwood formation includes living and thus respiring parenchyma cells. Functions of these parenchyma cells include storage and transport of NSCs (Plavcová & Jansen, 2015) and for mature trees that have been sampled, the average age of NSC carbon increases with distance from the cambium (Richardson *et al*., 2015; Trumbore *et al*., 2015). Therefore, respiration of older substrates deeper in the stem is expected. However, the large differences in both ^13^C and ^14^C between different sampling depths indicate that lateral exchange of gases is slow compared to rates of internal production/consumption. Although the potential metabolic activity of parenchyma cells presumably does not decline with age (i.e. stem depth) (Spicer & Holbrook, 2007b), overall respiration is expected to decline deeper into the stem due to lower oxygen concentration (Spicer & Holbrook, 2007a) and potentially also a lower percentage of live cells. Thus, total CO_2_ production rates should decline from the cambium to deeper inside the stem. In addition, very low diffusional velocities of gases through live, undried wood (Sorz & Hietz, 2006) are well known and provide an explanation for the accumulation of high concentrations of CO_2_ inside sapwood (Teskey *et al*., 2008).

There is an ongoing discussion about the origin of CO_2_ emitted from tree stems (Teskey *et al*., 2008) that we have to consider when interpreting the data from our stem chambers. It now is widely recognized that CO_2_ can be affected by various post‐respiratory processes including transport and refixation before it is emitted to the atmosphere (see Trumbore *et al*. (2013) for a summary). There are two important questions that have to be addressed here specifically: first, we have shown that ^14^C‐enriched CO_2_ can be found in all investigated tree stems, but contributes very little to surface emissions under normal conditions. Could this change in a girdled tree? Or in other words, could the observed increase in ^14^CO_2_ efflux simply be explained because the enriched in‐stem CO_2_ contributes an increasing portion to efflux with respiration presumably declining? Second, what is the role of CO_2_ produced elsewhere in the tree and being transported to the stem beneath the chamber, thus contributing to the chamber samples? Data from the live stem core incubations is very important in answering both these questions. For all three measurement dates, live stem core incubations confirm the radiocarbon data measured in chamber samples at the same time. In contrast to the complex situation described above, however, the stem cores should be devoid of any diffusional [CO_2_] gradients or transport effects, as they are no longer affected by sapflow and were allowed to equilibrate with the atmosphere for several hours before incubation. The cores thus should reflect current CO_2_ production by live tissue. The good agreement between the chamber and live stem core incubations thus allows two conclusions: first, the ^14^C‐enriched CO_2_ clearly originates from current respiration, not from increased diffusion of old CO_2_ that had been trapped in the stem; and second, although CO_2_ produced elsewhere (like e.g. in the roots) could add to the local emission beneath the chambers, this obviously has no effect on the local ^14^CO_2_ efflux. This makes sense, as we have to assume that all parts of the tree below the girdling are forced into mobilization of reserve C for survival.

Having identified current respiration as the origin for the ^14^C‐enriched CO_2_, we still have to discuss the possibility that this is not respiration of live tree cells, but could be fungal decomposition that the trees succumbed to as a consequence of weakened defenses. We consider this a highly unlikely explanation for the observed increase in Δ^14^CO_2_. First, all stem cores (including additional stem cores extracted in August 2014 for laboratory extractions) were visually inspected for signs of decomposition, and none was found. Second, the increase of ^14^C started immediately (i.e. within days) after girdling – it is highly unlikely that trees would succumb to fungal infestation this fast. Third, fungal decomposition of SC would result in the production of CO_2_ with the same isotopic signature as the decomposed tissue. With these trees being presumably older than 100 yr, they would have formed structural carbohydrates beginning in the pre‐bomb period and then all the way throughout the bomb‐spike, thus covering radiocarbon signatures between < 0 to almost 1000‰. Assuming that decomposition significantly contributed to the observed values, we thus would have to expect a much higher variability in the measured Δ^14^C, depending on how fast the decomposition progressed through the stem in the individual trees. Having said this, it still is possible for fungal decomposition to contribute locally at presumably low rates to the CO_2_ pool, we just find it highly unlikely that it is the dominant process behind the observed development of Δ^14^C over time.

The girdled trees survived for *c*. 10 months after girdling before showing clear signs of mortality. We did not measure growth or respiration during this time, but we have to assume that both were significantly reduced after girdling, which could explain why the CO_2_ concentrations at the end of the 24 h live stem core incubation declined between subsequent campaigns in the girdled trees, but remained stable in the control trees (Table 1). We are thus unable to draw any conclusions about the actual size of the reserve pools in these trees. However, it has been reported that NSC pools in trees contain enough C to rebuild the whole canopy of a mature tree between one to four times (Hoch *et al*., 2003; Würth *et al*., 2005), and tropical angiosperm trees in particular are known to have higher parenchyma fractions than temperate trees (Martínez‐Vilalta *et al*., 2016; Morris *et al*., 2016), and also were reported to have much higher starch concentrations than temperate or boreal trees (Martínez‐Vilalta *et al*., 2016), so it is not surprising that our trees survived for such a prolonged period and still were able to produce measurable amounts of CO_2_ during our sampling campaigns.

In summary, our results show that these trees have been able to build up reliable decade‐old storage C pools that they can access in times of stress. This mobilization follows a very clear chronology, with younger C being accessed first (last in, first out), suggesting that lateral mixing of the reserve pools is incomplete. Considering that the reserve pools integrate C from more than a decade, it is probable that these trees are reasonably well‐buffered against occasional periods of limited C assimilation.

## Author contributions

J.M., S.T., N.H. and N.K. planned the research; J.M. and N.K. designed the field experiment and conducted the field work; J.M. analysed the data; and J.M., S.T., N.H. and N.K. wrote the manuscript.
